# Muscle afferent ASIC3 upregulation mediates the exacerbated exercise pressor reflex in male rats following hindlimb ischemia–reperfusion

**DOI:** 10.14814/phy2.70457

**Published:** 2025-07-09

**Authors:** Lu Qin, Xuexin Zhang, Jianhua Li

**Affiliations:** ^1^ Heart and Vascular Institute The Pennsylvania State University College of Medicine Hershey Pennsylvania USA

**Keywords:** blood pressure, exercise, peripheral artery disease

## Abstract

We examined if the hindlimb muscle ischemia–reperfusion (IR) alters ASIC3‐mediated muscle afferent activity in regulating the exercise pressor reflex (EPR). APETx2 (an ASIC3 antagonist) was arterially injected into the hindlimb before static muscle contraction. The mean arterial pressure (MAP) and heart rate (HR) were recorded. ASIC3‐mediated MAP response was studied via intra‐arterially injected lactic acid (LA). Western blot and immunofluorescence were used to determine the ASIC3 expression and location in L4–6 dorsal root ganglion (DRG). Calcium imaging was applied to detect pH6.7‐induced Ca^2+^ entry in the isolated muscle DRG neurons. IR amplified the peak MAP response to muscle contraction (sham vs. IR: *p* = 0.031), which was reduced by the blockage of ASIC3 with APETx2 (baseline vs. blockage: *p* < 0.001). The peak MAP responses to LA were increased in IR rats (sham vs. IR, 1 μmol/kg: *p* < 0.05; 2 and 4 μmol/kg: sham vs. IR, *p* < 0.01) and were reduced by APETx2 (baseline LA control vs. blockage: *p* = 0.013). ASIC3 protein expression was increased in IR L4‐6 DRGs (sham vs. IR, *p* = 0.012). APETx2 attenuated the pH 6.7‐induced Ca^2+^ entry (ΔF340/F380: sham vs. IR, *p* = 0.017; IR vs. IR + APETx2, *p* = 0.003). Increased ASIC3 signaling amplifies muscle afferent activity and exacerbates the EPR following IR.

## Graphical abstract

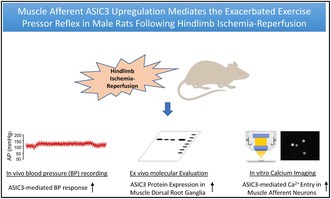

## 1 INTRODUCTION

Peripheral artery disease (PAD) is a significant cardiovascular condition that severely restricts blood flow to affected limbs. PAD patients frequently suffer from intermittent claudication (IC), characterized by a markedly reduced pain‐free walking distance, along with early‐onset exertion and ischemic muscle pain during daily activities (Gardner et al., 1999). This exercise intolerance condition is partly attributed to the hyperactive exercise pressor reflex (EPR), which has been observed in PAD patients (Muller et al., 2012; Stone & Kaufman, 2015). The EPR is a critical cardiovascular reflex that regulates blood pressure and sympathetic nerve activity during exercise. It is initiated by the mechano‐ and metabo‐receptor activation in muscle afferent nerves, which respond to muscle contraction and changes in the muscle interstitial environment (Kaufman & Forster, 1996; McCloskey & Mitchell, 1972). While lactic acid (LA) is a major metabolite produced in skeletal muscle during exercise, the protons accumulate and reduce the pH value within the muscle interstitial fluids. Acid‐sensing ion channels (ASICs) are a key group of metabo‐receptors in muscle afferent nerves that sense the protons. Among the six different ASIC subtypes (ASIC1a, 1b, 2a, 2b, 3, and 4), ASIC_3_ is predominantly located in the primary DRG neurons and opens as pH drops below ~7 (Sutherland et al., 2001). In ischemic muscles, the muscle interstitial pH value decreases to 6.7–7.0, while during maximal intensity exercise, it could be even lower when approaching exhaustion (Hermansen & Osnes, 1972; Wemmie et al., 2013). ASIC3 activation in muscle afferents enhances sympathetic outflow and cardiovascular responses, thereby exaggerating the EPR during ischemic conditions (Kim et al., 2020; Tsuchimochi et al., 2010). In using an animal model of femoral artery occlusion (FAO), previous studies indicate ASIC3 is effectively involved in hyperactive EPR in PAD (Kim et al., 2020; Tsuchimochi et al., 2010). It is notable that FAO is one of the most effective models used to mimic blood flow deficiency, amplified blood pressure (BP) responses, and reduced exercise performance in PAD patients (Colleran et al., 2010; Kuczmarski et al., 2018; Tsuchimochi et al., 2010). Besides impairing daily physical performance, a hyperactive EPR in PAD is linked to an elevated risk of cardiovascular diseases and events (Lewis et al., 2008).

In PAD, treatment often involves a combination of revascularization surgery on the femoral artery and supervised exercise interventions (Beckman et al., 2021; Fakhry et al., 2015). However, revascularization following FAO can lead to ischemia–reperfusion (IR) injury in reperfused tissues. Meanwhile, during IC, the arterial blood and oxygen supply cannot meet the demand of the exercising skeletal muscle (Pipinos et al., 2006). Patients often rest and wait for symptoms to subside before resuming activities. This cycle of rest and activity leads to repeated IR episodes in the skeletal muscle (McDermott, 2015). In line with the observation in the PAD patients, we have noted an exaggerated EPR in a rat hindlimb IR model over a time course of 18, 66, and 114 h post‐IR (Qin & Li, 2022), indicated by an increased BP response to static muscle contraction in decerebrated rats. Blood flow reperfusion is expected to normalize pH levels altered by lactate accumulation, potentially attenuating ASIC3 activation. The pathological milieu following IR, however, challenges this assumption. Using the same rat IR model (Qin & Li, 2022), we have identified that the activity of P2X3—a primary receptor for ATP in thin‐fiber muscle afferent DRG neurons—is heightened while regulating the EPR response in hindlimb IR (Qin et al., 2024). When the blood flow is resumed and the phosphocreatine pathway of ATP may contribute to the proton consumption (Broxterman et al., 2017), the breakdown of accumulative ATP during hydrolysis may still lead to the production of additional protons during exercise (Robergs et al., 2004). In addition, P2X3 is not the only receptor that is contributing to the exaggerated exercise pressor reflex in PAD (Butenas et al., 2023; Tsuchimochi et al., 2011; Yamauchi et al., 2013). This partly serves as a rationale for us to raise an important question: could ASIC3 also mediate the hyperactivated EPR observed post‐IR?

To explore this question, our study aimed to determine the role of ASIC3 in regulating the EPR following IR. We employed western blot analysis to measure ASIC3 expression in DRG. Additionally, we used whole animal preparations to assess whether the BP response to ASIC_3_ activation and static muscle contraction was reduced after blocking ASIC3 receptors in muscle afferent nerves. We also conducted calcium imaging studies to evaluate ASIC3‐mediated neuron activity in isolated muscle DRG neurons. We hypothesized that ASIC3 expression and activity in thin‐fiber muscle afferent nerves would be elevated in IR rats and that these exaggerated responses could be mitigated by blocking ASIC3.

## 2 METHODS

### 2.1 Ethical approval

All experimental procedures were approved by the Institutional Animal Care and Use Committee of Penn State College of Medicine (Protocol no.: PRAMS201147671) and were conducted per the National Institutes of Health Guide for the Care and Use of Laboratory Animals. Sprague–Dawley rats were housed in accredited temperature and ventilation‐controlled facilities with a 12:12 h‐light–dark cycle and ad libitum access to standard rat chow (Evigo Teklad Global 18% Protein Rodent Diet, Cat no. 2018) and water. A total of 60 Sprague–Dawley rats were used across all experimental procedures. The number of animals used for each experiment is also indicated in the respective protocols. For survival surgeries, rats were anesthetized by inhalation of 2–5% isoflurane in 100% oxygen; buprenorphine hydrochloride (0.05 mg/kg, subcutaneously) was administered before the surgery for post‐operative pain relief. At the end of each non‐survival experiment, animals were humanely euthanized by inhalation of high‐concentration isoflurane (>5%), followed by cardiac removal to ensure death.

### 2.2 Justification of animal usage

We highly recognize the importance of involving female animals in the studies. As we need to evoke the EPR by static muscle contraction, the muscle tension will be a key factor in determining the pressor response. Previous studies and our ongoing study (manuscript in preparation) have found that the muscle tension of female rats during ventral root stimulation averages ~300 g, which is lower than that observed in male rats, averaging ~600 g (Koba et al., 2012; Schmitt et al., 2006). In addition, estrogen is a key factor for the ASIC3 function in DRG, and female PAD patients are mainly elderly women, so the ovariectomy model will also need to be used. We, therefore, consider it more suitable to conduct separate studies on male and female rats. Several studies on female rats are currently underway by our team. Therefore, in the present study, surgical procedures and downstream experiments were performed in adult male rats (400–450 g).

### 2.3 Surgeries of hindlimb IR

To perform the surgery of the hindlimb IR (Qin et al., 2024), a ligature was placed tightly with a slipknot around the femoral artery ~3 mm distal to the inguinal ligament. After the femoral artery occlusion, the rats were removed from the anesthesia and kept in the surgery room for 6 h. Six hours later, anesthesia was introduced again, and the surgery site was reopened. Blood flow reperfusion was induced by releasing the slipknot to return blood flow into the femoral artery. Surgical forceps were used to gently massage the artery to assist in removing the blood clot and recovering the blood flow. The surgical wound was carefully closed after observing the blood flow enter the previously ligated femoral artery. The same procedures were followed to obtain sham controls except that a suture was placed below the artery, but the artery remained intact. Following the surgery, the animals were kept in the surgical room for 2–3 h for observation and then returned to the animal facility. Apart from the western blot analysis (detailed below), all the in vivo and in vitro experiments were performed 18 h after the end of the blood flow reperfusion.

### 2.4 Examination of the EPR and ASIC3‐mediated BP responses following IR

A Kopf stereotaxic unit was used to fix the head of the rats. The rats were anesthetized by inhalation and ventilated. The jugular vein and common carotid artery were cannulated, respectively, for fluid delivery, and a pressure transducer was connected to monitor arterial BP. HR was calculated beat to beat from the arterial pressure pulse. During the experiments, baseline BP and fluid balance were maintained with a continuous infusion of saline, and body temperature was maintained at ~37°C with an electric heating pad (Harvard Apparatus).

Decerebration was performed to eliminate anesthesia's effects on the reflex pressor response (Smith et al., 2001). Before the procedure, dexamethasone (0.2 mg, i.v.) was injected to minimize brain stem edema. A transverse section was made anterior to the superior colliculus and extending ventrally to the mammillary bodies, and then all tissues from the rostral to the section were removed (Smith et al., 2001). Following this procedure, the anesthesia was withdrawn, and a ventilator was applied to the rats. At least 60 min later, one of the following protocols was used to study the involvement of ASIC3 in the EPR following IR.

#### 2.4.1 Protocol 1: Blockage of ASIC3 during evoking the EPR by static muscle contraction following IR

A total of 20 rats were used for this protocol, divided into sham (*n* = 7) and IR (*n* = 13) groups. In each group, MAP, HR responses, and muscle tension were recorded during baseline, APETx2 blockade, and recovery phases. To study the reflex BP and HR responses during muscle contraction, the left hindlimb was used for sham/IR procedure, ventral root stimulation, and intra‐arterial injections. A laminectomy procedure was performed to expose the lower lumbar and upper sacral portions of the spinal cord and the peripheral ends of the transected L4 and L5 ventral roots. A platinum bipolar stimulating electrode was placed under the ventral root. The static muscle contractions were induced by electrical stimulation of the ventral roots (30 s, 3× motor threshold with a duration of 0.1 ms at 40 Hz). To block the ASIC_3_, its antagonist, APETx2 (Alomone Labs, Cat no.: STA‐160, 100 μg/kg, 0.1 mL) (Tsuchimochi et al., 2011), was dissolved in saline and injected through the femoral artery. The length of injection time was ~1 min, and the muscle contractions were induced immediately afterward. During this protocol, three sessions were performed: (1) pre‐blockage baseline control session: static muscle contraction only; (2) blockage session: APETx2 injection before static muscle contraction; and (3) post‐blockage recovery session: static muscle contraction only. The interval among sessions was ~30 min to ensure the recovery of the animal from the previous stimulation and the wash‐out of the injected reagents.

#### 2.4.2 Protocol 2: BP Responses by ASIC3 activation following IR

A total of 14 rats were used for this protocol, divided into sham (*n* = 6) and IR (*n* = 8) groups. MAP and HR responses were recorded during baseline, 1 μmol/kg LA, 2 μmol/kg LA, 4 μmol/kg LA, and recovery phase. To study the reflex BP and HR responses to ASIC3 activation following IR, both the left and right hindlimbs were used for sham or IR procedures and intra‐arterial injections, resulting in 12 tested limbs in the sham group and 16 tested limbs in the IR group. Specifically, the sham procedure was performed bilaterally in sham rats, while the IR procedure was performed bilaterally in IR rats. We chose this design rather than applying sham and IR procedures to opposite limbs within the same animal because the IR‐induced impact on unilateral hindlimb muscle could potentially affect the contralateral limb (Enko et al., 2011; Shenker et al., 2003; Song et al., 2012). Such systemic effects (e.g., inflammation) may alter ASIC3 expression or function, thereby confounding direct comparisons between IR and sham conditions within a single animal. Moreover, the ventral root stimulation technique used to evoke static muscle contraction in *Protocol 1* allows for contraction in only one leg at a time, necessitating separate groups for sham and IR procedures in this protocol to ensure methodological consistency. To activate muscle afferent ASIC3, ASIC3 agonist LA (1, 2, and 4 μmol/kg body weight, Sigma Aldrich, Cat no.: W261114), resolved in saline (0.1 mL), was given via the femoral artery. The concentration of LA was determined based on our previous study (Liu et al., 2010). A catheter (PE10) was inserted into the femoral artery for lactic acid delivery. The duration of the injection of LA was 1 min. BP responses to arterial injection of LA were examined in sham and IR rats.

#### 2.4.3 Protocol 3: Administration of LA with/without ASIC3 antagonist on BP responses following IR

A total of 7 rats were used for this protocol, divided into sham (*n* = 3) and IR (*n* = 4) groups. In each group, MAP and HR responses were recorded during baseline, APETx2 blockade, and recovery phases. Both the left and right hindlimbs were used for sham or IR procedures and intra‐arterial injections, yielding six tested limbs in the sham group and eight tested limbs in the IR group. BP responses were examined in three sessions in the following order: (1) pre‐blockage baseline control: arterial injection of 4 μmol/kg body weight LA; (2) blockage session: arterial injection of 4 μmol/kg body weight LA with APETx2 (100 μg/kg); and (3) post‐blockage recovery session: arterial injection of 4 μmol/kg body weight LA. The interval among sessions was ~30 min.

To restrict systemic washout and localize intra‐arterial injectates (e.g., APETx2 or LA) within the hindlimb circulation, in Protocols 2 and 3, the femoral artery was gently occluded for ~1 min immediately following injection using a pair of forceps pressed against the hindlimb proximal to the catheter insertion site. No venous snare was used due to the prone positioning of the animal and the anatomical inaccessibility of femoral veins in this setup.

### 2.5 Western blotting analysis

A total of nine rats were used for Western blot analysis, divided into Sham (*n* = 4 rats) and IR (*n* = 5 rats) groups. The L4–L6 DRGs were collected from both left and right sides of each animal, yielding a total of 8 DRG samples in the Sham group and 10 DRG samples in the IR group for protein quantification. Each DRG was processed and analyzed individually. As described previously (Qin et al., 2020), a total protein of rat L4—L6 DRG tissues in sham and IR limbs was extracted. Thirty microgram of protein was loaded in 10% Mini‐Protean TGX Precast gels (Bio‐Rad) after being boiled at 95°C for 5 min in SDS sample buffer, then electrophoretically transferred to polyvinylidene difluoride (PVDF) membrane. After blocking with 5% non‐fat milk in 0.1% Tween‐TBS buffer (TBST) for 1 hr., the membrane was incubated with rabbit anti‐ASIC_3_ (1:500, Alomone, Catalog no.: ASC‐018, RRID:AB_2039701) primary antibody at 4°C overnight and then incubated with HRP‐conjugated anti‐rabbit secondary antibody (1:2000, Cell Signaling Technology, Catalog no.: 7074, RRID:AB_2099233) at room temperature for 1 hr. Immunoreactivity was visualized using an enhanced chemiluminescence system (Cell Signaling Technology, Cat no.: 6883). The membrane was stripped and incubated with an anti‐β‐actin primary antibody (1:1000, Sigma Aldrich, Catalog no.: A5441; RRID:AB_476744) as the internal protein expression control. A pre‐stained protein ladder (Thermo Scientific, PageRuler™, Catalog no. 26616) was used. For visualization, bands between 10 and 180 kDa were recorded. The ASIC3 protein (~67 kDa) and β‐actin (~42 kDa) bands were quantified. The optical densities of targeted bands were analyzed using the NIH Image J Software.

### 2.6 Immunofluorescence

Two naïve (non‐surgical) rats were used for immunofluorescence procedure to determine the distribution of ASIC3 in DRG neurons (Lu et al., 2012; Xing et al., 2012; Xing et al., 2013). DRG tissue sections were first fixed with cold 4% paraformaldehyde (PFA) for 10 min. After fixation, sections were rinsed three times with PBST (PBS + 0.1% Tween‐20), followed by incubation in Tris‐buffered saline with 0.1% Triton X‐100 for 10 min and an additional two washes with PBST to remove residual buffer. DRG sections were then co‐incubated with primary antibodies to rabbit anti‐ASIC3 (1:200, Alomone, Catalog no.: ASC‐018, RRID:AB_2039701) and mouse anti‐peripherin‐fluorescein isothiocyanate (FITC) (1:200, Novus Biological, Catalog no.: NBP2‐73337F, RRID:AB_3386815), which labels small‐diameter unmyelinated C‐fiber/group IV afferents. After this, the sections were rinsed with PBST and incubated with anti‐rabbit secondary antibody conjugated with Alexa Fluor‐594 (1:250, Thermo Fisher, Catalog no.: A‐11012, RRID:AB_2534079). Following final washes, sections were mounted using Fluoromount‐G Mounting Medium (Fisher Scientific, Catalog no.: 00‐4958‐02) and coverslipped with Nunc™ Thermanox™ coverslips (Thermo Fisher, Catalog no.: 150067) for fluorescence imaging.

### 2.7 Labeling of hindlimb muscle afferent DRG neurons

Briefly as described (Li et al., 2020; Li et al., 2021), 4–5 days before sham and IR models were made, the lipophilic dye 1, 1′‐dioctadecyl‐3, 3, 3′, 3′‐tetramethylindocarbocyanine perchlorate (DiI, Thermo Fisher, Cat no.: D‐3911, 60 mg/mL) was injected into the superficial location (to ensure it would mainly be injected in white portion) of the gastrocnemius muscle. A total volume of 1 μL DiI tracer was administered at two to three different locations, with the needle left in the muscle for 1 min to ensure the full absorption of DiI. After that, the rats were returned to the facilities to wait for the fluorescent DiI retrograde to be transported to DRG to label muscle DRG neurons.

### 2.8 Calcium imaging in cultured muscle afferent DRG neurons

Muscle afferent DRG neurons were isolated from a total of eight rats, including four sham rats and four IR rats. As adapted from the protocols from our previous in vitro studies on muscle afferent DRG neurons, rats were anesthetized with over 5% isoflurane and then euthanized by the removal of the heart. L4–L6 DRGs were dissected and immediately transferred into ice‐cold Hank's balanced salt solution (HBSS). Under the dissecting microscope, the axons and connective tissue around the DRG were removed. The ganglia were then transferred into ice‐cold pre‐made cell culture medium [Dulbecco's Modified Eagle Medium (DMEM) (Gibco) with 10% FBS, 1% glutamine, and 1% penicillin–streptomycin], with the addition of collagenase Type D (Worthington, Cat no.: LS004186, 0.6 mg/mL; Roche) and trypsin (0.30 mg/mL; Worthington). The DRG tissue was minced thoroughly and then incubated at 34°C with shaking for 45 min. An equal volume of the cell culture medium was added to terminate the digestion process, and the mixture was centrifuged at room temperature for 5 min. The condensation was rinsed with HBSS twice. Then, the fresh cell culture medium was added, and the mixture was vortexed again to let the condensation dispense entirely in the medium. Filtered by a 40 μm Cell Strainer (Falcon®), the dissociated muscle afferent neurons, along with 2 mL of the above pre‐made DMEM medium, were then seeded on glass bottom peri‐dishes (Thermo Scientific, Cat no.: 12‐567‐401, Dia no. 35 mm) pre‐treated with laminin/poly‐l‐ornithine solution (Sigma, Cat no.: LPLO001). Then, the neurons were cultured at 37°C with 5% CO_2_ and 95% air in a cell culture incubator (VWR). The calcium imaging study was conducted at least 3 h later and completed within 24 h. Neurons from each surgical group were processed and imaged in separate experimental batches to avoid cross‐contamination or treatment bias. Neurons were clustered by condition and analyzed accordingly during calcium imaging.

During the calcium imaging experiment, the cells were cultured in a culture medium containing 4 μM Fura‐2‐acetoxymethyl ester (Fura‐2 am, Thermo Fisher, Cat no.: F1221) at 37°C with 5% CO2, 95% air in a cell culture incubator for 40 min. Cells were then rinsed three to four times and bathed in Ca^2+^‐4‐(2‐hydroxyethyl)‐1‐piperazineethanesulfonic acid (HEPES)‐buffered saline solution (Ca^2+^‐HBSS) (in mM: 140 NaCl, 1.13 MgCl_2_, 4.7 KCl, 2 CaCl_2_, 10 D‐glucose, and 10 HEPES, with pH adjusted to 7.4 with NaOH) for 5–10 min before Ca^2+^ is measured. Muscle DRG neurons were identified as Dil‐positive under an inverted microscope with a set of fluorescent filters (excitation: 550 nm, emission: 565 nm). The region of interest (ROI) was manually drawn around the soma of each DRG neuron using ImageJ. The number of pixels within each ROI varied depending on cell size, typically ranging from 50 to 200 pixels per neuron. Fluorescence images of several cells were recorded and analyzed with a digital fluorescence imaging system. Fura‐2 am fluorescence at an emission wavelength of 510 nm will be induced by excitation of Fura‐2 am alternately at 340 and 380 nm. The fluorescence ratio of 340 nm to that of 380 nm will be obtained on a pixel‐by‐pixel basis. For the Ca^2+^ entry measurement protocol, begin recording with 2 mM Ca^2+^‐HBSS (pH 7.4). At the 1st minute, switch to Ca^2+^‐free HBSS (pH 7.4); at the 2nd minute, switch to 2 mM Ca^2+^‐HBSS (pH 6.7); and at the 4th minute, switch back to Ca^2+^‐free HBSS (pH 7.4) to allow the recovery of the neurons for the next protocol. To observe Ca^2+^ entry following ASIC3 blockage, add 100 nM APETx2 along with 2 mM Ca^2+^‐HBSS (pH 6.7) following 1‐min baseline recording under Ca^2+^‐free HBSS (pH 7.4). Changes in intracellular calcium concentration (Δ[Ca^2+^]ᵢ) were assessed by calculating the change in the F340/F380 fluorescence ratio (ΔF340/F380), defined as the peak ratio value during the 60‐second stimulation period minus the average baseline ratio recorded during the 60‐second pre‐stimulation period. All experiments will be conducted at room temperature. A total of 11 neurons from Sham rats and 16 neurons from IR rats were recorded under the ASIC activation condition (pH 6.7). Of these, 8 neurons from the Sham group and 12 neurons from the IR group remained stable and viable for subsequent recording under ASIC3 inhibition (pH 6.7 + APETx2, 100 nM). Thus, each of these neurons was sequentially tested under both baseline and inhibitor conditions. The loss of neurons was due to unstable baseline signals or mechanical loss during reagent additions.

### 2.9 Statistical analysis

Unless specified, the data in this study were presented as the mean ± standard deviation (SD). SPSS for Windows, version 29.0, was used for all statistical analyses. To ensure that the data met the assumptions required for valid parametric analysis, normality and homogeneity of variance were assessed prior to using parametric statistical tests. When data violated assumptions of normal distribution or homogeneity of variance, appropriate nonparametric tests were applied (e.g., Mann–Whitney *U*‐test, Wilcoxon signed‐rank test). To avoid distortion of statistical results by extreme values, outliers were identified and removed using an interquartile range (IQR) method (values exceeding 1.5× IQR beyond the first or third quartile). Statistical analyses were conducted using independent t‐tests or two‐way ANOVA, as appropriate. An independent t‐test was used to compare ASIC3 protein expression between the Sham and IR groups. Two‐way ANOVA was applied to evaluate the effects of group (Sham vs. IR) and treatment/time (e.g., baseline, blockage, recovery) in physiological and calcium imaging experiments. When significant main effects or interactions were observed, simple effect analyses were performed using Bonferroni's post hoc test to assess differences between specific group combinations. Due to partial data loss across repeated measures (e.g., significant outliers and unstable recordings during each session), a non‐repeated measures two‐way ANOVA was chosen to maximize statistical power. For calcium imaging data that did not meet the assumption of normal distribution (assessed by the Shapiro–Wilk test), nonparametric tests were used. The Mann–Whitney *U*‐test was applied for unpaired comparisons between the Sham and IR groups. The Wilcoxon signed‐rank test was used for paired comparisons between sham and sham + APETx2 or IR and IR + APETx2 conditions. The sample size was determined using G*Power 3.1 to ensure adequate power for the statistical analyses. A *p* value less than 0.05 was considered statistically significant, with a statistical power greater than 0.80 to minimize the risk of Type II errors. Exact *p*‐values are provided in the results section and figure legends.

## 3 RESULTS

### 3.1 Animal grouping and data inclusion

All rats used in this study were weight‐matched prior to experimentation, with an average body weight of 418.44 ± 38.83 g in the Sham group (*n* = 26, including naive rats used for immunohistochemistry) and 410.00 ± 27.08 g in the IR group (*n* = 34) at the time of experimentation. No significant difference was found between sham and IR rats (*p* = 0.34). Across protocols, slight variations in sample size between experimental phases (e.g., baseline, blockade, recovery) occurred due to technical issues or the removal of statistical outliers. Outliers were excluded only if they met predefined statistical criteria based on the interquartile range (IQR >1.5×) during normality assessment. In rare instances, recovery‐phase data could be salvaged even when blockade‐phase data were lost, resulting in a slightly higher sample size for recovery than for blockade.

### 3.2 Blockage of ASIC3 during static muscle contraction in sham and IR decerebrated rats

**FIGURE 1:**
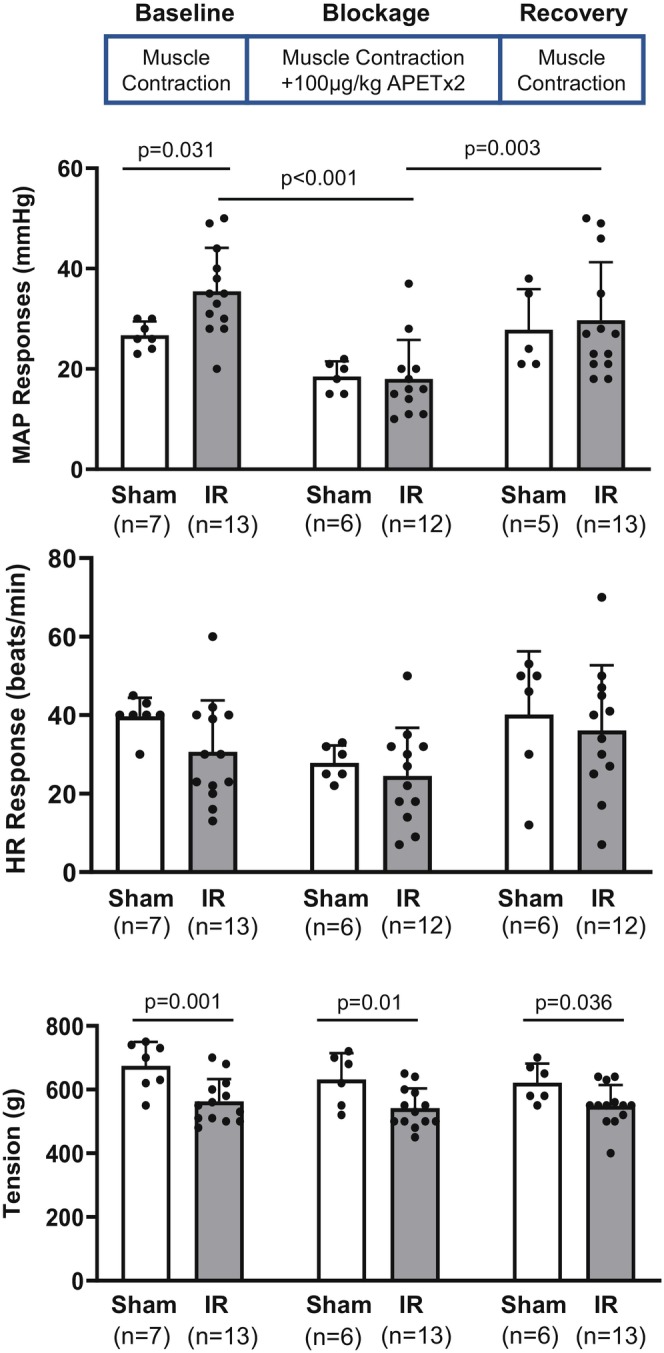
Blockage of ASIC3 during evoking exercise pressor reflex (EPR) in the sham and IR rats. The EPR was evoked by a protocol of ~30s static muscle contraction, with a transductor continuously monitoring the muscle tension during the contraction. Mean arterial pressure (MAP) and heart rate (HR) were recorded. During each experiment, three sessions were designed: (1) Baseline control: Static muscle contraction was performed without arterial injection of ASIC3 antagonist, APETx2; (2) Blockage: Static muscle contraction was performed following the arterial injection of APETx2 (100 μg/kg); and (3). Recovery: Static muscle contraction was performed after the APETx2 was washed out. The interval among sessions was ~30 min. The white bars represent the sham group, and the gray bars represent the IR group. Two‐way [group (sham vs. IR) × time (baseline vs. blockage vs. recovery)] ANOVA was used to test the differences among groups. Upper panel: MAP responses to static muscle contraction in sham and IR rats. Compared with the sham group, the MAP response in IR rats was amplified during the baseline control (*p* = 0.031). In IR rats, the blockage of ASIC3 with APETx2 significantly reduced the MAP responses, and the reduction was reversed during the recovery session (baseline vs. blockage, *p* < 0.001; and blockage vs. recovery, *p* = 0.003). Middle panel: HR responses to static muscle contraction in sham and IR rats. Lower panel: Muscle tension during the muscle contraction. There was no significant difference among the baseline, blockage, and recovery within both sham and IR rats. The muscle tension was lower in IR rats than in sham rats. Data were presented as mean ± standard deviation (SD). The round dots in the figure bar indicate individual data points in the respective group. The sample size was indicated in the bracket below the group name. Minor variations in sample size between phases occurred due to occasional loss of valid recordings or exclusion of statistical outliers based on interquartile range criteria (IQR >1.5×). Exact *p* values were shown in the figure.

No significant difference was found between sham and IR groups regarding the basal MAP and HR in each session (*Protocol 1* in Table 1). The main effect of time was found significant [*F*_(2,56)_ = 10.905, *p* < 0.001] regarding the MAP responses to muscle contraction. Specifically, the blockage of ASIC3 with APETx2 significantly reduced the peak MAP responses (baseline vs. blockage: 35 ± 9 mmHg, *n* = 13 vs. 18 ± 8 mmHg, *n* = 12, *p* < 0.001) in IR rats. The reduction was reversed during the post‐blockage recovery session (blockage vs. recovery, 18 ± 8 mmHg, *n* = 12 vs. 30 ± 12 mmHg, *n* = 13, *p* = 0.003). Although the overall main effect of group was not statistically significant (*F*_(1,56)_ = 1.950, *p* = 0.169), phase‐specific post hoc analysis revealed that compared with the sham group, the peak MAP response in IR rats was amplified during the pre‐blockage baseline control session (sham vs. IR: 26 ± 3 mmHg, *n* = 7 vs. 35 ± 9 mmHg, *n* = 13, *p* = 0.031). This suggests that the group‐dependent response is conditionally expressed, likely due to the MAP responses in the sham and IR groups not being differentiated during the blockade phase. This also suggests that ASIC3 in both sham and IR was successfully blocked by the dosage of APETx2 used in the present study. From a physiological perspective, these findings align with the known amplified muscle afferent activities in PAD (Colleran et al., 2010; Kim et al., 2020; Kuczmarski et al., 2018; Qin et al., 2024; Qin & Li, 2022; Tsuchimochi et al., 2010). No significant difference was found among groups regarding the peak HR response [*F*_(1,50)_ = 2.274, *p* = 0.138]. The muscle tension was lower in IR rats than in sham rats (baseline sham vs. IR, 674 ± 75 g, *n* = 7 vs. 563 ± 70 g, *n* = 13, *p* = 0.001; blockage sham vs. IR, 631 ± 82 g, *n* = 7 vs. 541 ± 62 g, *n* = 13, *p* = 0.01; and recovery sham vs. IR, 621 ± 60 g, *n* = 7 vs. 549 ± 65 g, *n* = 13, *p* = 0.036) (Figure 1).

**TABLE 1:** Summary table of basal mean arterial pressure (MAP) and heart rate (HR) during each whole‐animal experiment protocol.

Protocol 1	Pre‐blockage baseline		Blockage		Post‐blockage recovery	
	MAP (mmHg)	HR (bpm)	MAP (mmHg)	HR (bpm)	MAP (mmHg)	HR (bpm)
Sham	101 ± 21	456 ± 63	105 ± 18	480 ± 53	109 ± 19	444 ± 46
IR	104 ± 16	469 ± 91	101 ± 17	463 ± 62	103 ± 15	480 ± 62

Protocol 2	Control		1 mM		2 mM		4 mM		Recovery	
	MAP (mmHg)	HR (bpm)	MAP (mmHg)	HR (bpm)	MAP (mmHg)	HR (bpm)	MAP (mmHg)	HR (bpm)	MAP (mmHg)	HR (bpm)
Sham	105 ± 21	397 ± 66	106 ± 25	421 ± 87	103 ± 18	401 ± 65	104 ± 18	424 ± 81	103 ± 19	434 ± 69
IR	108 ± 17	379 ± 66	106 ± 19	372 ± 63	112 ± 10	381 ± 85	109 ± 15	405 ± 84	109 ± 12	406 ± 91

Protocol 3	Pre‐blockage baseline		Blockage		Post‐blockage recovery	
	MAP (mmHg)	HR (bpm)	MAP (mmHg)	HR (bpm)	MAP (mmHg)	HR (bpm)
Sham	115 ± 13	389 ± 76	109 ± 19	400 ± 50	112 ± 22	403 ± 73
IR	96 ± 13^a^	348 ± 21	96 ± 19^a^	350 ± 30	93 ± 11^a^	341 ± 26^a^

*Note*: Protocol 1: Blockage of ASIC3 during evoking the EPR by static muscle contraction following ischemia–reperfusion (IR); Protocol 2: BP responses by ASIC3 activation following IR; Protocol 3: Administration of LA with/without ASIC3 antagonist on BP responses following IR. Data are presented as mean ± standard deviation (SD).

^a^ Versus sham, *p* < 0.05. Exact *p* values were described in the manuscript text.

### 3.3 ASIC3‐mediated MAP responses in sham and IR decerebrated rats

**FIGURE 2:**
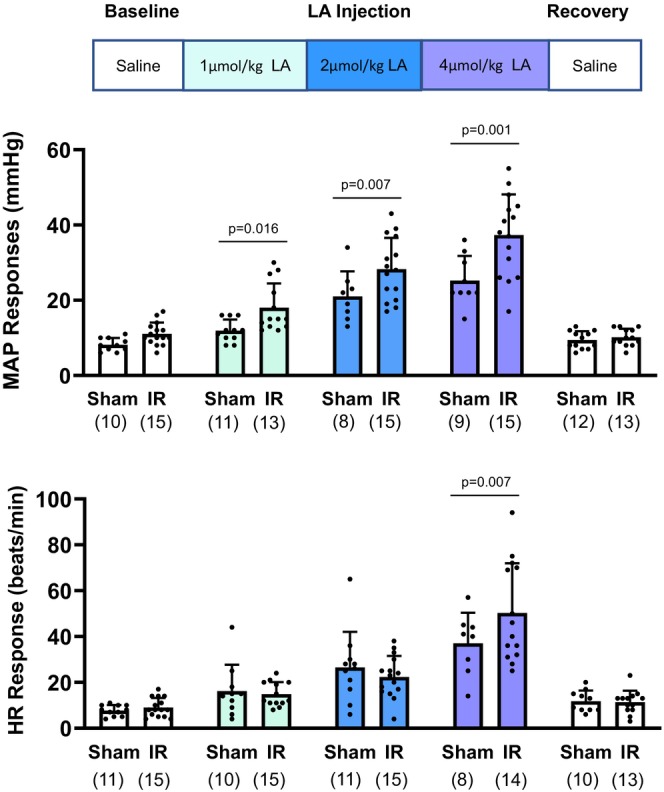
MAP response to lactic acid (LA) injection in the sham and IR rats. Different doses of LA (1, 2, and 4 μmol/kg body weight) were arterial injected into the hindlimb to activate ASIC3 in the muscle afferent terminals of the decerebrated rats. A total of 12 limbs from 6 Sham rats and 16 limbs from 8 IR rats were tested. Mean arterial pressure (MAP) and heart rate (HR) were recorded. Before and after the injection of LA, vehicle solution, 0.9% saline was injected as baseline and recovery control. As indicated in the figure, the white color bar indicates saline control during the baseline and recovery session. The light blue color bar indicates 1 μmol/kg body weight LA injection, the dark blue color indicates 2 μmol/kg body weight LA injection, and the purple color indicates 4 mM LA injection. Two‐way [group (sham vs. IR) × time (baseline vs. LA vs. recovery)] ANOVA was used to test the differences among groups. Compared with the sham, the MAP response to LA was higher in IR rats, regardless of the LA concentration. Except for 4 μmol/kg LA injection, no significant difference was found between sham and IR regarding the HR responses to LA injection. Data were presented as mean ± standard deviation (SD). The round dots in the figure bar indicate individual data points in the respective group. The sample size was indicated in the bracket below the group name. Minor variations in sample size between phases occurred due to occasional loss of valid recordings or exclusion of statistical outliers based on interquartile range criteria (IQR >1.5×). Exact *p* values were shown in the figure.

No significant difference was found between sham and IR groups regarding the basal MAP and HR in each session (*Protocol 2* in Table 1). The main effects of group and time were found significant [Group: *F*_(1,42)_ = 6.925, *p* = 0.012; Time: *F*_(2,42)_ = 10.905, *p* < 0.001] regarding the MAP response to LA injection. Compared with the sham, the peak MAP responses to LA injection were significantly increased in IR rats (1 μmol/kg LA: sham, 12 ± 3 mmHg, *n* = 11 vs. IR, 18 ± 6 mmHg, *n* = 13, *p* = 0.016; 2 μmol/kg: sham, 21 ± 7 mmHg, *n* = 8 vs. IR: 28 ± 8 mmHg, *n* = 15, *p* = 0.007; 4 μmol/kg: sham, 25 ± 7 mmHg, *n* = 9 vs. IR: 37 ± 11 mmHg, *n* = 15, *p* < 0.001). The main effect of group was found significant [Group: *F*_(1,41)_ = 4.271, *p* = 0.046] regarding the HR response to LA injection. No significant difference was found among the HR responses to LA injection in sham and IR following 1 and 2 μmol/kg LA injection (1 μmol/kg: sham, 16 ± 11 bpm, *n* = 10 vs. IR: 15 ± 5 bpm, *n* = 15, *p* = 0.759; and 2 μmol/kg: sham, 26 ± 15 bpm, *n* = 11 vs. IR: 22 ± 9 bpm, *n* = 15, *p* = 0.343). There was a significant difference in the HR responses to 4 μmol/kg LA injection (4 μmol/kg: sham, 37 ± 13 bpm, *n* = 8 vs. IR: 50 ± 22 bpm, *n* = 14, *p* = 0.007) (Figure 2).

**FIGURE 3:**
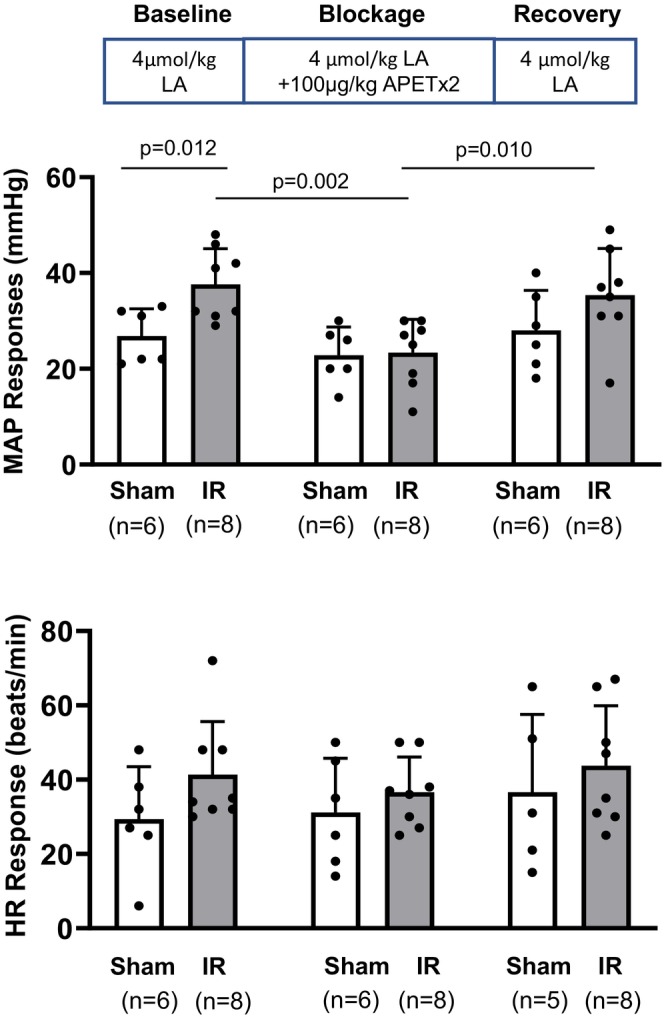
Blockage of ASIC3 during lactic acid (LA) injection in the sham and IR rats During each experiment, three sessions were designed: (1) Baseline control: LA (4 μmol/kg body weight) was injected without arterial injection of APETx2 (100 μg/kg); (2) Blockage: LA was injected following the arterial injection of APETx2 (100 μg/kg); and (3) Recovery: LA was injected after the APETx2 was washed out. Upper panel: MAP responses to LA injection in sham and IR rats. Two‐way [group (sham vs. IR) × time (baseline vs. blockage vs. recovery)] ANOVA was used to test the differences among groups. Compared with the sham group, the MAP response in IR rats was amplified during the baseline control (*p* < 0.05). In IR rats, the blockage of ASIC3 with APETx2 significantly reduced the MAP responses (baseline vs. blockage, *p* < 0.01). Lower panel: HR responses to LA injection in sham and IR rats. No significant difference was found among groups (all *p* > 0.05). Data are presented as mean ± standard deviation (SD). The round dots in the figure bar indicate individual data points in the respective group. The sample size was indicated in the bracket below the group name. Minor variations in sample size between phases occurred due to occasional loss of valid recordings or exclusion of statistical outliers based on interquartile range criteria (IQR >1.5×). Exact *p* values were shown in the figure.

Apart from the post‐blockage recovery session (sham vs. IR: 403 ± 73 bpm, *n* = 6 vs. 341 ± 26 bpm, *n* = 8, *p* = 0.022), no significant difference was found between sham and IR groups regarding the basal HR (sham vs. IR in baseline control and blockage, *p* = 0.127 and 0.062, respectively) (*Protocol 3* in Table 1). Compared with the baseline control, APETx2 significantly reduced the peak MAP response to LA in IR rats (baseline LA control, 38 ± 7 mmHg, *n* = 8 vs. blockage, 23 ± 7 mmHg, *n* = 8; *p* = 0.002), and the reduction was reversible as evidenced by the increased peak MAP to LA injection after the washout of APETx2 (ASIC3 blockage, 23 ± 7 mmHg, *n* = 8 vs. post‐washout recovery, 35 ± 10 mmHg, *n* = 8; *p* = 0.010). No significant difference was found among the HR responses in three conditions [baseline LA control vs. ASIC3 blockage vs. post‐washout recovery: 41 ± 14 bpm, *n* = 8 vs. 36 ± 9 bpm, *n* = 8 vs. 44 ± 16 bpm, *n* = 8; *F*_(2,35)_ = 0.465, *p* = 0.632]. The APETx2 had no effect on the LA‐induced peak MAP and HR responses in sham rats (peak MAP responses: baseline LA control vs. blockage vs. recovery, 27 ± 6 mmHg, *n* = 6 vs. 23 ± 6 mmHg, *n* = 6 vs. 28 ± 8 mmHg, *n* = 6; *F*_(2,36)_ = 0.763, *p* = 0.474; HR responses: baseline LA control vs. blockage vs. recovery: 29 ± 14 bpm, *n* = 6 vs. 31 ± 14 bpm, *n* = 6 vs. 37 ± 21 bpm, *n* = 5; *F*_(2,36)_ = 0.919, *p* = 0.408) (Figure 3).

### 3.4 ASIC3 protein expression and ASIC3‐mediated Ca^*2*+^ entry in isolated muscle afferent DRG neurons

**FIGURE 4:**
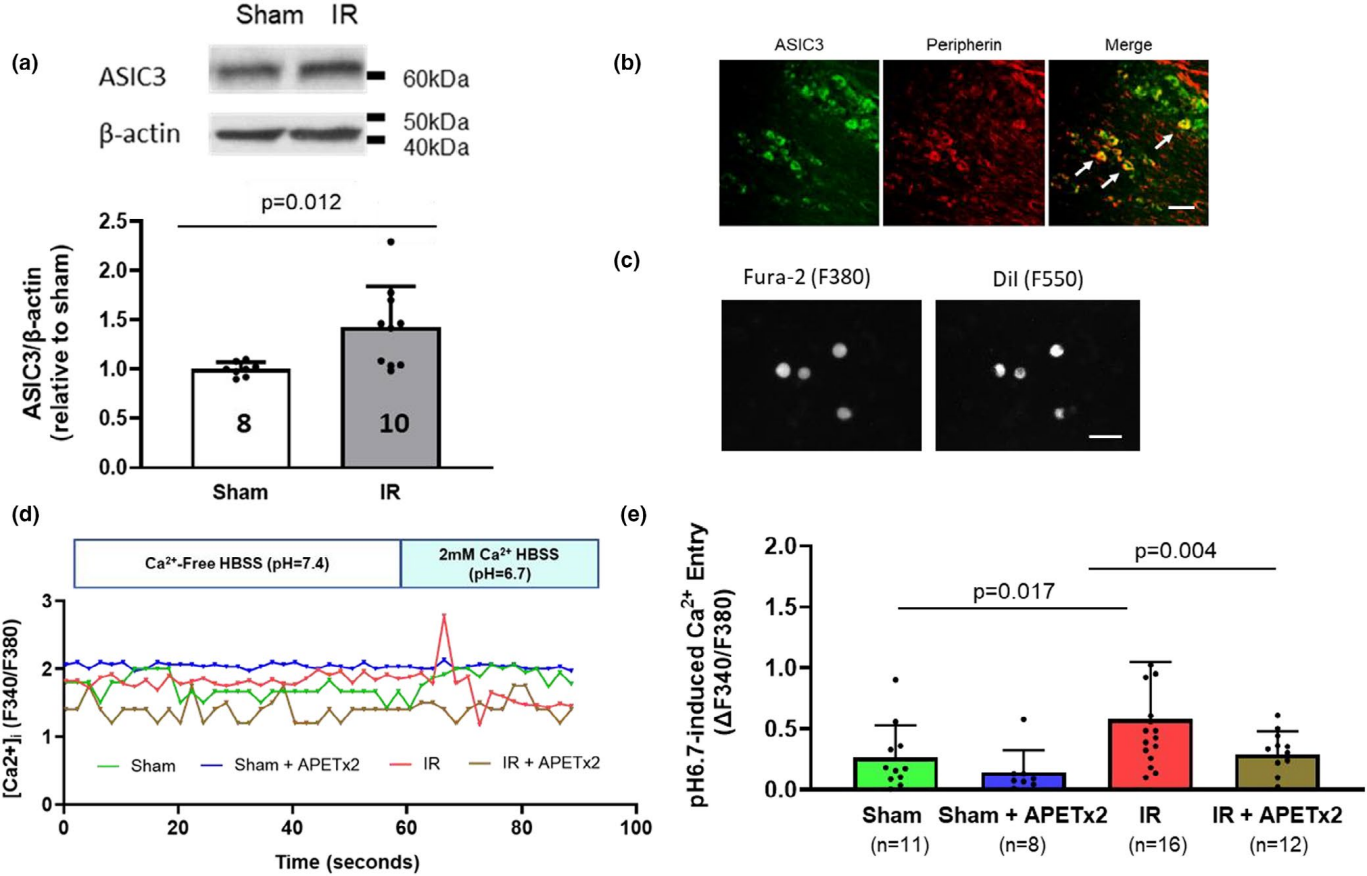
Delineating the role of ASIC3 in L4‐6 DRG of sham and IR rats by ex vivo and in vitro studies. (a) Western blot detected the protein expression of ASIC3 in the protein extraction of L4‐6 DRG. The optical density was normalized by the β‐actin protein expression in the respective samples. Western blot quantification of ASIC3 expression in L4–L6 DRGs (Sham: *n* = 8 DRGs from 4 rats; IR: *N* = 10 DRGs from 5 rats). An independent t‐test was applied to analyze the difference between the sham and IR groups. The dark bar indicates the sham group, and the gray bar indicates the IR group. Compared with sham (*n* = 8), the ASIC3 protein expression was higher in L4‐6 DRG of IR rats (*n* = 10) (*p* = 0.012). The molecular band indicates that ASIC3 was detected near the band of 60 kDa (molecular weight ~67 kDa), and β‐actin was detected near the band of 40 kDa (molecular weight ~42 kDa). The numbers in the data bar indicate the sample size of each group. (b) Co‐localization of the ASIC3 and peripherin (marker of C‐fiber) in rat L4‐6 DRG. Upper panel: ASIC3 (Green) and co‐expressed with peripherin (red). The white arrows in the merged pictures indicate the location of the neurons co‐expressed with ASIC3 and peripherin (yellow). The bar in the right low corner indicates 100 μm. (c) Identification of muscle afferent DRG neurons during calcium imaging study. Pictures were taken with the black‐and‐white colored camera under the microscope. Experiments were performed in neurons that detected both 380 nm (F380, upper panel) and 550 nm (F550, lower panel) wavelengths, indicating those neurons were positive with DiI injected to the gastrocnemius. The bar in the right low corner indicates 100 μm. (d) Representative trace of the calcium imaging experiment in isolated muscle afferent DRG neurons sham and IR rats with and without APETx2. Green line indicated the Ca^2+^ entry induced by pH 6.7 in muscle afferent DRG neuron of sham rats; Blue line indicates Ca^2+^ entry induced by pH 6.7, with the addition of APETx2, in muscle afferent DRG neuron of sham rats; Red line indicates the Ca^2+^ entry induced by pH 6.7 in muscle afferent DRG neuron of IR rats; Golden line indicates Ca^2+^ entry induced by pH 6.7, with the additional of APETx2, in muscle afferent DRG neuron of IR rats. (e) Ca^2+^ entry is indicated by the ratio of F340 and F380. The Mann–Whitney *U*‐test was applied for unpaired comparisons between the Sham and IR groups. The Wilcoxon signed‐rank test was used for paired comparisons between sham and sham + APETx2, or IR and IR + APETx2 conditions. The green bar indicates sham group, blue bar indicates sham + APETx2, red bar indicates IR group and brown bar indicates IR + APETx2. Compared with sham (*n* = 11), the Ca^2+^ entry to pH 6.7 was higher in IR group. The APETx2 attenuated thus effect. Data are presented as mean ± standard deviation (SD). The round dots in the figure bar indicate individual data points in the respective group. The sample size was indicated in the data bar. Exact *p* values were shown in the figure.

Following the in vivo experiments, we performed ex vivo and in vitro experiments to test the hypothesis that the alternation of ASIC3 in muscle afferent DRG is attributed to the EPR response in IR. As shown in Figure 4a, western blotting in the L4‐6 DRG shows that, compared with sham rats, there was a significant increase in ASIC3 protein expression in L4‐6 DRGs of IR rats (sham vs. IR: 1.00 ± 0.07, *n* = 8 vs. 1.42 ± 0.41, *n* = 10, *p* = 0.012). In addition, the expression of ASIC3 protein was found in peripherin‐positive L4‐6 DRG neurons (Figure 4b). In the in vitro setting, muscle afferent DRG neurons were isolated from the L4‐6 DRG and calcium imaging was used to test calcium entry to the intracellular space from extracellular solution (Figure 4c). Compared with the muscle afferent DRG neurons isolated from the sham rats, the Ca^2+^ entry induced by pH 6.7 solution was significantly increased in the neurons from the IR rats (ΔF340/F380: sham vs. IR, 0.19 ± 0.16, *n* = 10 vs. 0.51 ± 0.29, *n* = 13; *p* = 0.017, Mann–Whitney *U*‐test). Furthermore, APETx2 (100 nM) significantly attenuated the Ca^2+^ entry in muscle afferent neurons isolated from IR rats (ΔF340/F380: IR vs. IR + APETx2, 0.51 ± 0.29, *n* = 16 vs. 0.28 ± 0.21, *n* = 12; Z = −2.845, *p* = 0.004, Wilcoxon signed‐rank test). In contrast, APETx2 had no significant effect on Ca^2+^ entry in DRG neurons from sham rats (ΔF340/F380: sham vs. sham + APETx2, 0.51 ± 0.29, *n* = 11 vs. 0.28 ± 0.21, *n* = 8; Z = −1.540, *p* = 0.123, Wilcoxon signed‐rank test) (Figure 4d,e).

## 4 DISCUSSION

In the present study, key findings include the following: (1) IR amplified EPR, which was significantly attenuated following the ASIC3 blockage by APETx2 (Figure 1); (2) IR enhanced ASIC3‐mediated BP response with LA injection (Figure 2), which could be reversed by ASIC3 blockage by APETx2 (Figure 3); (3) Compared with the sham, there was an increased ASIC3 protein expression in L4‐6 DRG (Figure 4a); (4) ASIC3 protein was expressed in peripherin‐positive DRG neurons (Figure 4b); and (5) There was an amplified pH 6.7‐induced Ca^2+^ entry in isolated muscle afferent DRG neurons following IR. By blocking ASIC3, the pH 6.7‐induced Ca^2+^ entry was attenuated in the muscle afferent DRG neurons from IR rats (Figure 4d,e). These findings have significant clinical implications for PAD patients, particularly those undergoing revascularization procedures. IR may sensitize ASIC3 signaling in muscle afferents, contributing to heightened cardiovascular responses and limiting exercise capacity. Targeting ASIC3 could provide a novel therapeutic avenue for managing the amplified exercise‐induced BP response in this population.

ASIC3 is a key mediator in regulating EPR, with signals primarily originating from group III and IV muscle afferents. Previous studies showed either ASIC3 genetic knockout or pharmacological ASIC3 blockage using APETx2 has no effect on the blood pressure (BP) response to static muscle contraction in a non‐ischemic hindlimb (Kim et al., 2019; Tsuchimochi et al., 2011). However, both genetic knockout and pharmacological blockage of ASIC3 attenuate the heightened BP responses to muscle contraction in an ischemic hindlimb with FAO (Kim et al., 2020; Stone et al., 2015; Tsuchimochi et al., 2011). In the present study, APETx2 injection significantly decreased BP responses to muscle contraction in IR rats. While there was a tendency, the APETx2 did not significantly decrease the BP response to muscle contraction in sham‐operated controls. In the present study, the muscle tension during the contraction was lower than that in the sham group. This might be attributed to muscle function or muscle mass decline following the IR‐induced muscle damage, as previous studies have shown that even short durations of IR (e.g., 30 min to 3 h of ischemia followed by 1–3 h of reperfusion) can significantly alter the morphological and biochemical properties of the hindlimb muscles (de Carvalho et al., 2023). As we did not assess muscle weight, fiber composition, or perform direct muscle function tests in the current IR model, the relationship between muscle mass and tension remains speculative. Notably, previous studies have shown that higher muscle tension—often associated with greater exercise intensity—can lead to increased blood pressure (BP) and heart rate (HR) responses during muscle activity (Mitchell et al., 1983; Williamson et al., 2006). Our observation showed that the BP response to muscle contraction was increased in IR despite a reduction in muscle tension, which further proves our hypothesis that IR amplified the EPR independently of absolute muscle force. Future studies will incorporate muscle weight and fiber‐type analysis to further dissect the mechanical and structural contributions to altered muscle function in this model.

To specifically study the metabo‐reflex involvement of ASIC3 in regulating the BP response, LA was administered to simulate the increased LA accumulation during exercise. However, it should be noted that LA solution has a pH around 3 and will stimulate the entire ASICs family, particularly acid‐sensing ion channel 1a (ASIC1a) (Ducrocq et al. 2020), transient receptor potential cation channel subfamily V member 1 (TRPV1) (Ugawa et al. 2002) and might also trigger the release of inflammatory by‐products such as cyclooxygenase, arachidonic acid, and bradykinin. Therefore, APETx2 was applied to inhibit ASIC3. By doing so, we can ensure the BP response is from ASIC3 but not other mediators. The results in the present study indicate that the BP response to different concentrations of LA injection (1, 2, and 4 μmol/kg) was consistently increased in IR rats (Figure 2), while the amplified BP response to the 4 μmol/kg LA injection was significantly reversed when APETx2 was injected prior to the LA injection (Figure 3). In the ex vivo studies by western blot, we observed that the protein expression of ASIC3 was increased in L4‐6 muscle afferent DRG (Figure 4a), which was consistent with a previous study by Queme et al. showing the upregulated ASIC3 mRNA expression in T1–4 DRG of a forelimb IR mice model (Queme et al., 2020). Although ASIC3 expression was found to increase in both ischemic and ischemia–reperfusion (IR) conditions, the underlying mechanisms differ. In one of our previous studies, we assessed the time‐course response of ASIC3 expression in the L4‐6 dorsal root ganglia (DRG) following femoral artery occlusion (FAO). Our results indicated that ASIC3 expression began to increase at 24 h post‐FAO, but not at 6 h. Therefore, in the current study, the increase in ASIC3 expression is likely driven by factors beyond ischemia alone. Ross et al. (2016) demonstrated that ASIC3 in sensory neurons plays a key role in the pathological process of musculoskeletal pain following forelimb IR, mediated by pro‐inflammatory cytokines such as interleukin‐1β (IL‐1β). Genetic knockout of the IL‐1β receptor suppressed ASIC3 expression. Although we did not directly assess pro‐inflammatory cytokines in the present study, it is reasonable to hypothesize that they may be one of the primary factors contributing to the upregulation of ASIC3 in muscle DRG neurons following hindlimb IR. Using a decerebration approach to largely eliminate the influence of central command, we were able to focus specifically on the exercise pressor reflex (EPR) and evaluate blood pressure (BP) and heart rate (HR) responses following hindlimb IR. Our results suggest that ASIC3 plays a unique role in regulating BP responses to exercise, likely upregulated after the induction of pro‐inflammatory cytokines following IR.

One also should be aware of the complexity of variables involved during the in vivo and ex vivo studies. Despite deliberately controlling the manner during the reagent injection in the blood supply of the gastrocnemius muscle, ASIC3 has been known to be expressed not only in muscle afferents but also in other cells and tissues (Li & Xu, 2011). Therefore, in vitro studies were necessary to investigate the isolated muscle afferent DRG neuron to ensure it is the neuron itself, not other components within the DRG (e.g., glial cells, vascular smooth muscle cells) that play a role during the external stimuli. In one of our previous in vitro studies (Li et al., 2023), we used whole cell patch clamp to hold the membrane potential at −70 mV to specifically study the ASIC currents, including ASIC1a and ASIC3, in rat models of FAO/IR. The conclusion from that study highlights that the ASIC3 current is a critical player when the ASIC currents are amplified following FAO/IR, which paves the way for our current study to focus on the role of ASIC3 in regulating the EPR following IR. Following the patch clamp study, we still need to understand the role of ASIC3 in regulating neuron excitability when the cell membrane is intact, which is crucial for studying and understanding network‐level responses within the dorsal root ganglia or muscle‐innervating pathways. Therefore, in the present study, we utilized calcium imaging to measure muscle afferent neuron excitability during which ASIC3 was activated by the manipulation of the external pH, with or without the ASIC3 blockage. Calcium imaging offers significant advantages in measuring muscle afferent neuron excitability, particularly in investigating the cellular mechanisms of pain and sensory processing (Cai et al., 2020; Gold & Gebhart, 2010; Steele & Penhune, 2010). This technique enables the real‐time visualization of intracellular calcium fluctuations, a key indicator of neuronal activity, with high spatial and temporal resolution. Therefore, for muscle afferent neurons, whose excitability reflects their role in modulating sympathetic and cardiovascular responses, calcium imaging would be able to provide a direct measure of changes in response to specific stimuli, including pH shifts, that are physiologically relevant in conditions like ischemia or exercise (Kim et al., 2019; Kim et al., 2020; Stone et al., 2015; Tsuchimochi et al., 2010; Tsuchimochi et al., 2011). The other importance of looking at the Ca^2+^ mobility in the muscle afferent neurons is that this measurement also indicates the communication impact of the primary sensory DRG neurons to the secondary neurons in the dorsal horn of the spinal cord. While the current setting of our present study is in vitro instead of directly observing the inter‐neuron communication in the in vivo condition, we benefited from the in vitro setting by evaluating the real‐time Ca^2+^ mobility when the reagents are directly applied in the extracellular environment of the muscle afferent neurons. As a part of the EPR, the muscle afferent DRG neuron terminals detect the signals from the interstitial space of the skeletal muscle, which excites the neuron and initiates a cascade of events that promotes neurotransmitter (e.g., glutamate and substance P) release and is critical for activating dorsal horn neurons (Hunt & Mantyh, 2001). In states of high neuronal excitability, such as during sustained muscle contraction or ischemia, increased Ca^2+^ entry in the intracellular space can lead to prolonged or intensified signaling to the dorsal horn neurons, thereby amplifying the EPR (Meyer, 1989). As a part of our future efforts, an in vivo calcium imaging setting will be established to observe the neuron activation during static muscle contraction and the injections of different reagents. In combination with our ongoing findings from the in vitro calcium imaging experiments, those approaches will be of great importance to add novel information on the subset of activated DRG neurons during evoking EPR.

When the ASIC3 is activated by extracellular acidosis, it primarily allows sodium ions (Na^+^) to enter the cell, leading to membrane depolarization. While the activation of ASIC3 does not directly carry or be permeable to Ca^2+^, the depolarization can in turn activate voltage‐gated calcium channels (VGCCs) and therefore influence intracellular Ca^2+^ levels through this indirect mechanism by facilitating the opening of VGCCs (Liu et al., 2015). Once activated, VGCCs open and allow Ca^2+^ entry into the intracellular space. In the present study (Figure 4d,e), we found that the Ca^2+^ entry induced by pH 6.7 stimuli was significantly higher in muscle afferent neurons isolated from the IR rats than in those from sham rats. With the co‐administration of APETx2 and pH 6.7 solution, the heightened Ca^2+^ entry in IR rats was blocked. This tendency is mainly consistent with what we observed in a whole animal study. While the Ca^2+^ entry measurement is one of the endpoints of the current studies, it will initiate our future exploration regarding how the calcium channels and which subtypes are involved during different stimuli on the muscle afferent neurons. Calcium channel‐related medications are considerably safer and have a more prolonged effect than those working on sodium or potassium channels due to the specific role calcium plays in cellular processes, allowing for a more targeted therapeutic window with fewer side effects, and making them more favorable in elderly patients (Basile, 2004; Benetos et al., 2019). Therefore, PAD patients would benefit from those research findings regarding the safe medication options for their BP and cardiovascular health management. That will be one of the main directions for our future studies.

## 5 CONCLUSION

Taking the key results of the present study together, we conclude that ASIC3 in muscle afferents plays a crucial role in regulating EPR following IR. This sheds light on developing therapeutic targets in the BP management of PAD patients.

## AUTHOR CONTRIBUTIONS

Q.L. designed, performed, and supervised the experiments, analyzed and interpreted the results, generated figures, and drafted and revised the manuscript for submission. X.X.Z. contributed to experimental design and execution, data analysis and interpretation, and assisted in proofreading the manuscript and figure legends. J.H.L. oversaw the experimental design and execution, interpreted the results, and contributed to manuscript revision and proofreading.

## Data Availability

The datasets generated and analyzed during the current study are available from the corresponding authors upon reasonable request.
